# How the Brain Represents Language and Answers Questions? Using an AI System to Understand the Underlying Neurobiological Mechanisms

**DOI:** 10.3389/fncom.2019.00012

**Published:** 2019-03-12

**Authors:** Marco A. P. Idiart, Aline Villavicencio, Boris Katz, César Rennó-Costa, John Lisman

**Affiliations:** ^1^Department of Physics, Institute of Physics, Federal University of Rio Grande do Sul, Porto Alegre, Brazil; ^2^Department of Theoretical Informatics, Institute of Informatics, Federal University of Rio Grande do Sul, Porto Alegre, Brazil; ^3^School of Computer Science and Electronic Engineering, University of Essex, Colchester, United Kingdom; ^4^Computer Science and Artificial Intelligence Laboratory, Massachusetts Institute of Technology, Cambridge, MA, United States; ^5^Digital Metropolis Institute, Federal University of Rio Grande do Norte, Natal, Brazil; ^6^Volen Center for Complex Systems, Brandeis University, Waltham, MA, United States

**Keywords:** theta-gamma code, episodic memory, short-term (working) memory, memory retrieval, question and answer

## Abstract

To understand the computations that underlie high-level cognitive processes we propose a framework of mechanisms that could in principle implement START, an AI program that answers questions using natural language. START organizes a sentence into a series of triplets, each containing three elements (subject, verb, object). We propose that the brain similarly defines triplets and then chunks the three elements into a spatial pattern. A complete sentence can be represented using up to 7 triplets in a working memory buffer organized by theta and gamma oscillations. This buffer can transfer information into long-term memory networks where a second chunking operation converts the serial triplets into a single spatial pattern in a network, with each triplet (with corresponding elements) represented in specialized subregions. The triplets that define a sentence become synaptically linked, thereby encoding the sentence in synaptic weights. When a question is posed, there is a search for the closest stored memory (having the greatest number of shared triplets). We have devised a search process that does not require that the question and the stored memory have the same number of triplets or have triplets in the same order. Once the most similar memory is recalled and undergoes 2-level dechunking, the sought for information can be obtained by element-by-element comparison of the key triplet in the question to the corresponding triplet in the retrieved memory. This search may require a reordering to align corresponding triplets, the use of pointers that link different triplets, or the use of semantic memory. Our framework uses 12 network processes; existing models can implement many of these, but in other cases we can only suggest neural implementations. Overall, our scheme provides the first view of how language-based question answering could be implemented by the brain.

## Introduction

Although neuroscience has made progress in understanding how brain networks can execute simple tasks, this is not the case for the network basis of high-level tasks such as language processing. Thus, we do not yet understand, at the level of neural circuits, the required computations and how they might be neurally implemented (Barsalou, 2017). In an attempt to make progress in this direction, this paper addresses this challenge proposing algorithmic-level mechanisms that could be involved in implementing language processing in the brain, focusing on the process of question answering. We took an artificial intelligence program (START) that can answer some types of questions (Katz, 1988, 1997) and sought to identify neurally plausible processes by which this program could be implemented. The START system deals with natural language, stores information in memory, and uses this information to answer questions. We are aware that language and ultimately semantic processing involves complex interactions of different parts of the brain (Simmons et al., 2008), and areas of the brain beyond the classical language areas may be recruited when answering requires reasoning or reliving of experiences. Here, however, we consider the kind of processing that is restricted to simple recollection of facts stored using a limited set of linguistic cues, namely the information of the events and participants described in a sentence. In terms of the Dual Semantic Theory (Paivio, 1990) it could correspond to what is called a shallow semantic process. We believe that if we could neurally implement START, we could gain insight into the neural computations required for a high-level cognitive task. Such computations might be implemented by known types of neural networks. Alternatively, a valuable outcome would be the identification of computations not previously implemented. This identification would provide motivation to develop a greater repertoire of neural network computations as a basis of understanding high-level tasks (Berwick et al., 2013). A successful neural implementation might be incorporated into neuromorphic chips, providing a fundamental change in the methods for computerized question answering. In addition understanding how aspects of language can be neurally implemented can provide us with insights about how the language capacity emerged and evolved (Berwick et al., 2013; Hauser et al., 2014).

## Results

**Figure 1:**
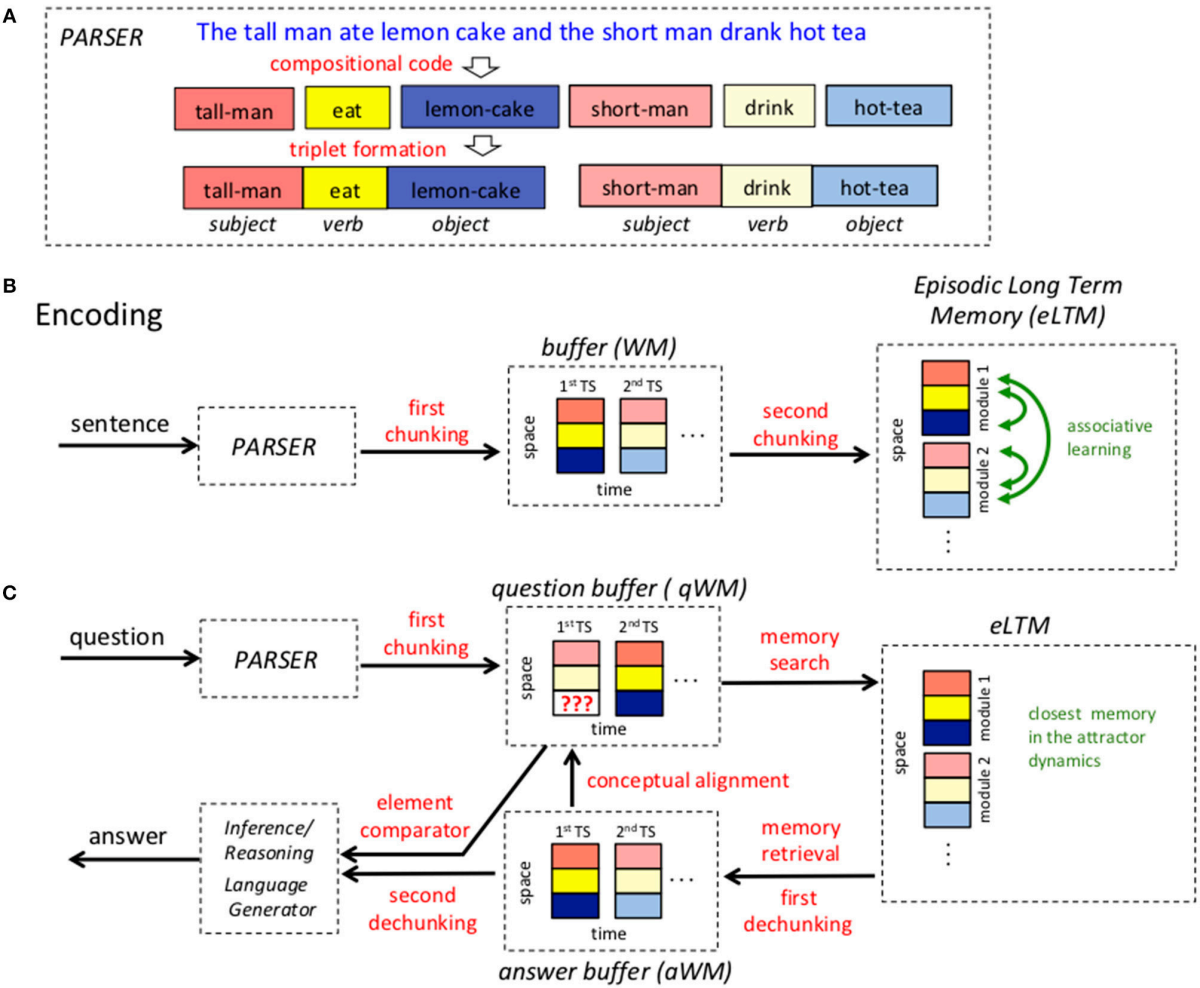
High level architecture of the model. **(A)** An example of a sentence represented as triplets. Stopwords are removed, adjectives/adverbs are incorporated using a compositional code and triplet roles are assigned. **(B)** The process of storing a sentence in episodic long term memory (eLTM). A complex sentence representing the episode is parsed and the triplet elements determined (subject,verb,object). The elements of the triplets are then chunked into a spatial pattern (vertical arrangement). The ordered triplets that encode a sentence are then stored in theta-gamma working memory (WM) buffer. Then, in a second chunking operation, all the triplets that represent a sentence are formed into a single spatial pattern in eLTM. **(C)** The process of answering a question. Words are first parsed into triplets. Triplets are chunked into spatial patterns that are stored in the question memory buffer (qWM). Each triplet in qWM is used to probe the eLTM, resulting in the selection and activation of the stored memory that most closely matches the question. This memory is retrieved and stored temporarily in the answer WM buffer (aWM). The contents of the qWM and aWM are dechunked, aligned and sent to an element comparator that looks for information in aWM to complete missing values (blanks) in qWM. The result is sent to an inference/reasoning module and finally to a language generator.

The proposed system for question answering has many components. The processing of an input sentence starts with a parser that recognizes parts of speech and converts language into a brain code, Figure 1A. Incoming information is then held in a working memory (WM) buffer. The key property of working memory is that information is actively represented by cell firing patterns, a process that need not involve modification of synaptic strengths. It is now clear that such buffers can actively store several items; indeed, one goal of our work is to suggest how such a buffer might encode a whole sentence. Information is then sent to an episodic long-term memory (eLTM) network where it is encoded by changes in synaptic weights, Figure 1B. Such networks thus need not display activity to keep information storage. The term “episodic” refers to memories with temporal and contextual information about events, the kind conveyed in a sentence (Tulving, 1985). It also refers to the fact that such networks can learn online, storing a sentence even after a single presentation. When a question is posed, it is similarly processed into a question working memory buffer (qWM), not with the goal of encoding it into long-term memory, but of eliciting from long-term memory the stored memory that contains the answer to the question posed. This memory is put into an answer WM buffer (aWM), dechunked and then compared to the question, thereby generating the information necessary to produce an answer, Figure 1C. Finally, there is a language generator that converts brain code back into natural language. In this initial effort, we have chosen to model the parser and the language generator as black boxes (see Hale, 2014; Stewart et al., 2014 for neurally plausible parsers), to concentrate on the neural implementation of processes more directly involved in question answering. Aspects of semantic memory useful in relating a query to stored memories are also not dealt with here. In the sections below we give a fuller description of each step. In a later part of the paper, we discuss how each of the processes might be neurally implemented.

### The Parser

A question answering system like START begins by parsing a sentence which involves recognizing parts of speech and determining syntactic functions (Chomsky, 1965; Briscoe, 1997; Harabagiu et al., 2003; Berwick et al., 2013). A critical step is the formation of triplets to represent relations among entities (Katz, 1988) composed of three elements, subject, verb, and object (also referred to as agent, predicate, and patient Frankland and Greene, 2015, or Arg1, Pred, Arg2; Baker et al., 1998; Baldwin et al., 2004, depending on the particular linguistic theory), which we assume are the elementary units of understanding (Baroni and Lenci, 2010), Figure 1A. Indeed there is evidence from fMRI that the left superior temporal sulcus, a region linked to sentence processing, might be subdivided into subregions or slots that represent these three elements (Frankland and Greene, 2015; Dehaene et al., 2018). The triplets are also reminiscent of the treelets that have been proposed for representing sentence fragments (Marcus, 2013).

An example of the use of triplets is

**<John, eat, cake>**

to represent the sentence “*John ate the cake*.” We indicate triplets by angle brackets with the elements separated by commas. For simplicity, we exclude stopwords (i.e., extremely common words that appear to have little semantic content, like articles) and verb tenses from the triplets. We assume parsing rules produce the same representation for similar sentences, even if they have some degree of syntactic variation (e.g., *John ate cake* and *The cake was eaten by John* are both represented by <John,eat,cake>). A more complex sentence may need additional operations and triplets. START has a list of prescriptions for dealing with complex sentences (Katz, 1988) that we will explain as the text progresses. Some of them are adopted in this work and some are modified in favor of a more neurally realistic mechanism.

### A First Chunking Stage Allows an Entire Sentence to Be Held in Working Memory

Initially language is processed as an ordered (temporal) sequence, and in our model we assume that it will ultimately be stored as a spatial pattern (i.e., without explicit temporal structure). To achieve this conversion of temporal information to spatial information, there are two stages of a process termed chunking. In the first chunking step, the three elements of a triplet are chunked into a single spatial activity pattern that is held in a working memory buffer. Recall that in such buffers, information is held by the pattern of actively firing cells. However, this pattern can have subdivisions. We assume that the working memory network has three subdivisions, one for the subject, one for the verb, and one for the object. This is the first chunking operation shown in Figure 1B. Note that when spatial patterns are represented, subdivisions of the spatial pattern are shown to be vertically offset.

A sentence may contain many phrases, requiring many triplets. We make the critical assumption that, for downstream processing, a sentence has to be stored as a whole in working memory. Although there is an ongoing debate on the mechanisms behind working memory (see the companion papers, Constantinidis et al., 2018; Lundqvist et al., 2018) here we adopt the view that multiple items of information are held in working memory in a buffer based on temporal multiplexing scheme organized by theta and gamma oscillations, as proposed some years ago by two of the authors of this paper (Lisman and Idiart, 1995). One of the reason for this is the recent and very compelling evidence from intracranial recordings of the human brain cortex where subjects performed a Sternberg-type task with lists of letters (Bahramisharif et al., 2018). The data show that regions tuned to specific letters display preferred theta phase of activation according to the letter serial position in the list. This added to similar data in the hippocampus (Heusser et al., 2016) demonstrates that the theta-gamma phase code might be a general coding scheme for WM. Such putative buffers encode single items within a gamma cycle; the multiple gamma cycles within a theta cycle allow the temporally compact (~100 mec) representation of up to ~7 items in defined order (Miller, 1956; Sternberg, 1966; Heusser et al., 2016). Thus, we assume that up to 7 triplets could be rapidly represented serially within the 7 gamma cycles (or gamma time-slots) of a theta cycle. The compactness of this representation allows the complex information in a sentence to be rapidly processed and communicated to other structures including the long-term memory network.

In START, a long sentence with 20 words might require up to 10 triplets. This would be too many triplets to store within the 7 cycles of a theta cycle. To give the model the capability of dealing with such sentences, we propose that two or more words can be stored within a triplet element, where content words like verbs and nouns correspond to the core semantic elements, but can be further described by modifiers like adjectives and adverbs. This process, that we refer to as compositional coding (Figure 1A) leads to an enriched type of word representation (a compositional word, or cWORD). With the greater representational compactness enabled by the compositional code, it is possible to store sentences using fewer triplets than START and thereby to neurally represent even very complex sentences.

To give a specific example, while in START descriptors are encoded by separate triplets (e.g., “*John ate a nice meal”* is encoded as two connected triplets: “ <*John,eat,meal*_1_> <*meal*_1_*,is,nice*>”), compositional coding yields a more economic scheme in which adjectives and adverbs are encoded together with the noun or verb they describe, see Figure 1A. Thus, in the example above, only 1 triplet would be required as the other would be incorporated into “meal” forming the concept “nice-meal” (we represent cWORDS by words connected by “-”):

**<John,eat,nice-meal>**

Indeed there is evidence the brain doesn't use actual words for storing episodes in long memory but instead uses semantic encoding. Such encoding scheme is supported by the finding that, even though subjects can recall verbatim a just-heard sentence, if some delay or interpolated material exists between presentation and testing subjects only retain its meaning (Sachs, 1967; Marcus, 2013). An important benefit of semantic encoding is that synonyms that differ greatly in their phonetics are similar semantically. This is important for question answering because questions may well be posed using synonyms of the word involved in the stored memory (e.g., *man* in the question and *guy* in the memory). Moreover, it is important to note that while the use of enriched words is likely to be feasible for adjective and adverbs describing inherent properties of nouns and verbs, it probably can't work for descriptive phrases (e.g., *the man with the red hat*) because the phrase may contain information very different from any of the inherent features of these words.

In summary, a combination of chunking (to arrange words into triplets) and compositional coding allows even long sentences to be represented during a single theta cycle (Figure 1A).

### A Second Chunking Stage Allows a Spatial Pattern in Long-Term Memory to Store a Sentence

We now come to the question of how the temporally coded information in the WM buffer is stored in eLTM and how this can be done in such a way that a sentence can be recalled given a cue that is anywhere in the stored sequence. This involves the second chunking operation. We propose that the network that encodes eLTM has many *modules*, each specialized to encode the triplet contained within a given gamma cycle (Figure 1B). Such modular organization is ubiquitous in the cortex (Lundqvist et al., 2010). Thus, the first eLTM module is devoted to the first triplet, the second module devoted to the second triplet, etc. The network has additional specializations that divide each module into 3 subregions, one for the subject, one for the verb, and one for the object. In this way the 21 elements of 7 triplets can be stored in a network having 21 subregions. This overall architecture of the eLTM network allows the temporal sequence of triplets to be laid out as a modular *spatial pattern*, thus achieving the second *chunking operation*.

Within this spatial organization, neurons become linked by synaptic contacts, thereby encoding the sentence as the association of words contained in it (de Almeida et al., 2007; Lundqvist et al., 2010; Rennó-Costa et al., 2014). The particular information about a cWORD is encoded by the spatial pattern of active cells within a subregion. Hebbian synaptic plasticity connects the different cells within an element, the different elements within a module, and the different modules. As a result of these network-wide connections, all aspects of the sentence become associated. Importantly, this reduction of a complete sentence to a spatial code allows standard network attractor properties to produce sentence recall, even when cued by a part in the middle or end of the sequence, e.g., not by the memory content of initial module (see next section). In summary, the short-range and long-range connections within the eLTM network have weights that collectively encode a sentence, even one of considerable complexity.

### Retrieving From Episodic Long-Term Memory: Finding the Memory Most Related to the Question

We next consider how the eLTM is searched during question answering. The goal of the search is to find the most similar stored memory, which is the one that may contain the missing information being sought in the query. Even though it is possible to describe an episode in many different ways using natural language, we assume the parser produces a single canonical sequence of triplets for each episode, that we call simply a sentence. We propose that when the question is posed, it is first parsed into a sequence of triplets and held in a *question WM buffer* (qWM), again organized by theta-gamma oscillations, Figure 1C. Then all eLTM modules are queried *in parallel* by the first triplet in the question buffer. If a module has that triplet in its synaptic memory the neural ensemble corresponding to that triplet is persistently activated. This is followed by a similar query using the second triplet in the question buffer, and so on, until all the triplets in qWM have been used in this way. Different sentences, however, can share triplets, and if they do, these triplets are usually in different modules. Therefore, the process of retrieving a sentence from eLTM is bound to be a process that can suffer from interference of competing putative memories, especially when triplets are searched in parallel. To mitigate interference of other memories we consider that inter-module connections can prime associated triplets in other modules that belong to the same memory. Priming puts target triplets into an activatable state should they receive subsequent input from the qWM buffer, but importantly, priming is not sufficient by itself to activate a triplet. After all triplets in qWM have been searched, some of the modules in eLTM will contain active triplets corresponding to a memory and these may be linked to primed triplets in other modules. By using priming, we guarantee that a triplet that is not in the query is never activated until the end of the search, reducing the crosstalk to a minimum. After all the triplets available in the question are used, a second type of interaction in the eLTM selects the memory that is closest to the query. In this phase, synaptic connections between modules and within modules are modulated so that they actually excite targets rather than merely priming them. The network goes to a global attractor state representing a single sentence, which corresponds to the best answer.

**Figure 2:**
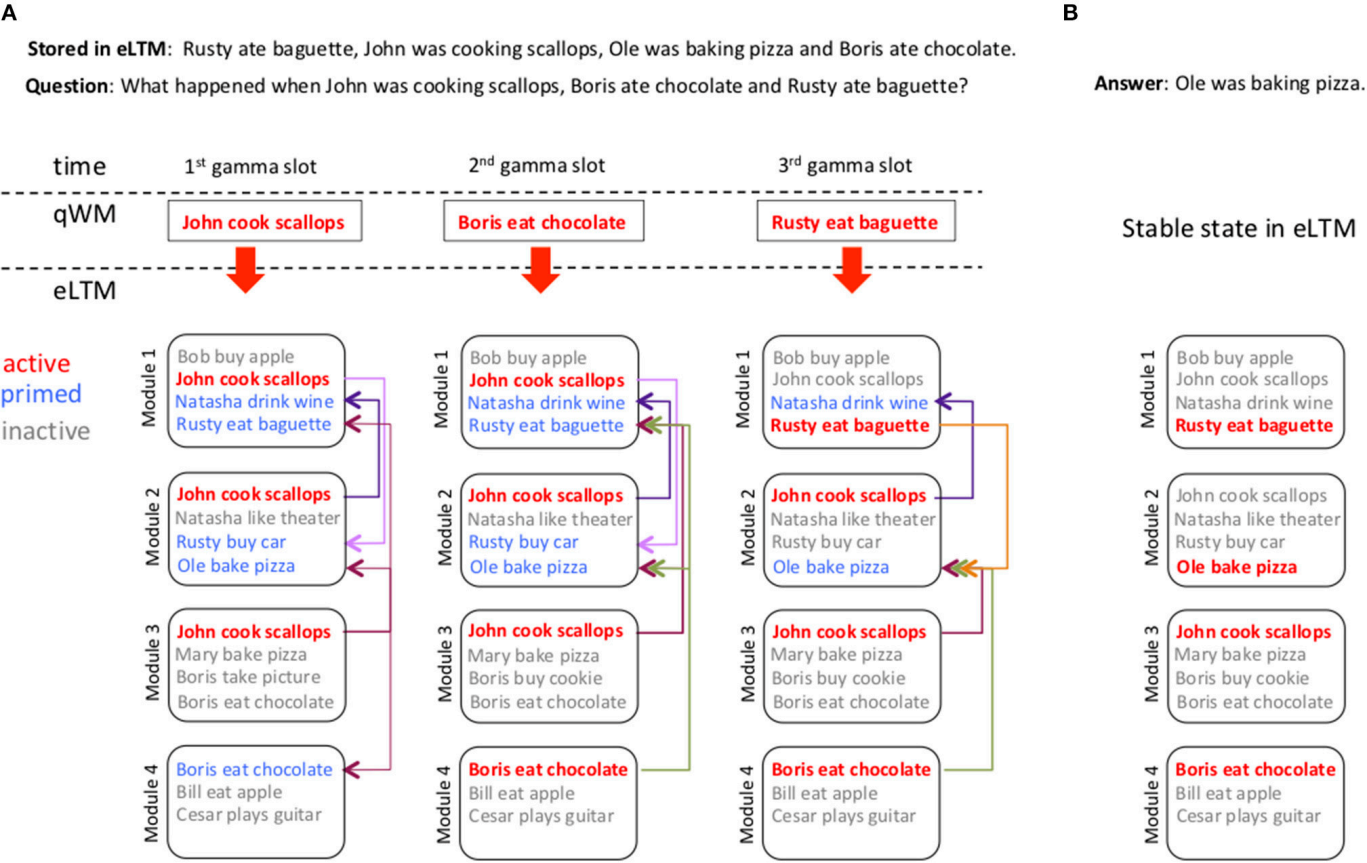
Probing of eLTM using the information in qWM. **(A)** Displays the process of answering the question “What happened when John was cooking scallops, Boris ate chocolate and Rusty ate baguette?” using the information “Rusty ate baguette, John was cooking scallops, Ole was baking pizza and Boris ate chocolate.” stored in eLTM among other possible overlapping memories. qWM is represented by a single rectangle with the triplet expression it holds in a specific gamma cycle slot (represented between the dashed lines). eLTM has 7 modules (only 4 shown), and each module has a list of triplet expressions that indicates its stored memories. Active memories are represented in red, primed memories in blue, and inactive memories in gray. Due to competition arising from inhibition a module can only hold one active memory, but can have many primed memories. The recurrent connections indicate the priming pathways stimulated by an active memory. If a target memory is already active we do not show the priming pathway. 1st gamma slot: The triplet held in the first gamma slot (John cook scallops) is searched in parallel in all modules activating the corresponding memories. 2nd gamma slot: The second triplet (Boris eat chocolate) is searched in eLTM. It does not replace the existing active memory in module 3 because this memory has been primed by the active memory in module 4. 3rd gamma slot: The last triplet is searched. It replaces an existing attractor in module 1, and does not interfere with the activity in other modules. **(B)** Displays the final state in eLTM. Once all triplets from qWM are searched the excitatory synaptic connections between modules and the inhibitory synaptic connections (not shown) within modules are simultaneously activated. eLTM thus becomes a full attractor neural network that retrieves a single stored sentence (the closest memory).

Figure 2 shows the sequence of stages of the retrieval process of the query: “What happened when John was cooking scallops, Boris ate chocolate and Rusty ate baguette?” in the context where among many stored information there is the memory “Rusty ate baguette, John was cooking scallops, Ole was baking pizza and Boris ate chocolate.” The first three panels show the temporal querying of eLTM. A different triplet is used at each gamma cycle to probe eLTM. If a given module has this particular triplet among its stored memories it becomes active (represented by red fonts), Figure 2A. A triplet activated in a given module in turn primes associated triplets in other modules (primed triplets are represented by blue fonts). Arrows indicate the intermodule synaptic connections used for priming. Gray fonts represent inactive triplets (triplets that were not in the queries but are stored in the module). As a new triplet from the question is added to the search process it can replace previously activated triplets in a module if it has been primed (for instance in Figure 2A, 3rd gamma slot, 1st module, the triplet “ < Rusty,eat,baguette>” replaces “ <John,cook,scallop>”). At the end of the search eLTM will have modules with active triplets and a series of primed triplets that are not active. In the final stage (Figure 2B), the recurrent connections are modulated so there are no longer primed triplets, they are either active or inactive, and the full memory is recovered. An important aspect of the parallel search is that the order of triplets in the query buffer need not match their order in eLTM, thereby providing the ability to deal with queries in which the word order doesn't match that of the stored memory.

### The Best Answer Is Dechunked and PUT Into the aWM Buffer

Once the best answer is retrieved, to enable comparison with the question held in qWM, this spatial pattern is partially dechunked so that the different triplets in the best answer are stored in different gamma slots of the answer WM buffer (aWM). This buffer, like qWM, uses theta-gamma coding to hold a sequence of triplets. Specifically, the triplet in the first module of eLTM is copied in the first gamma cycle to the aWM buffer, and so on. This is the first dechunking operation needed for question answering.

### A Comparator Operates on the qWM and aWM Buffers, Element by Element

**Figure 3:**
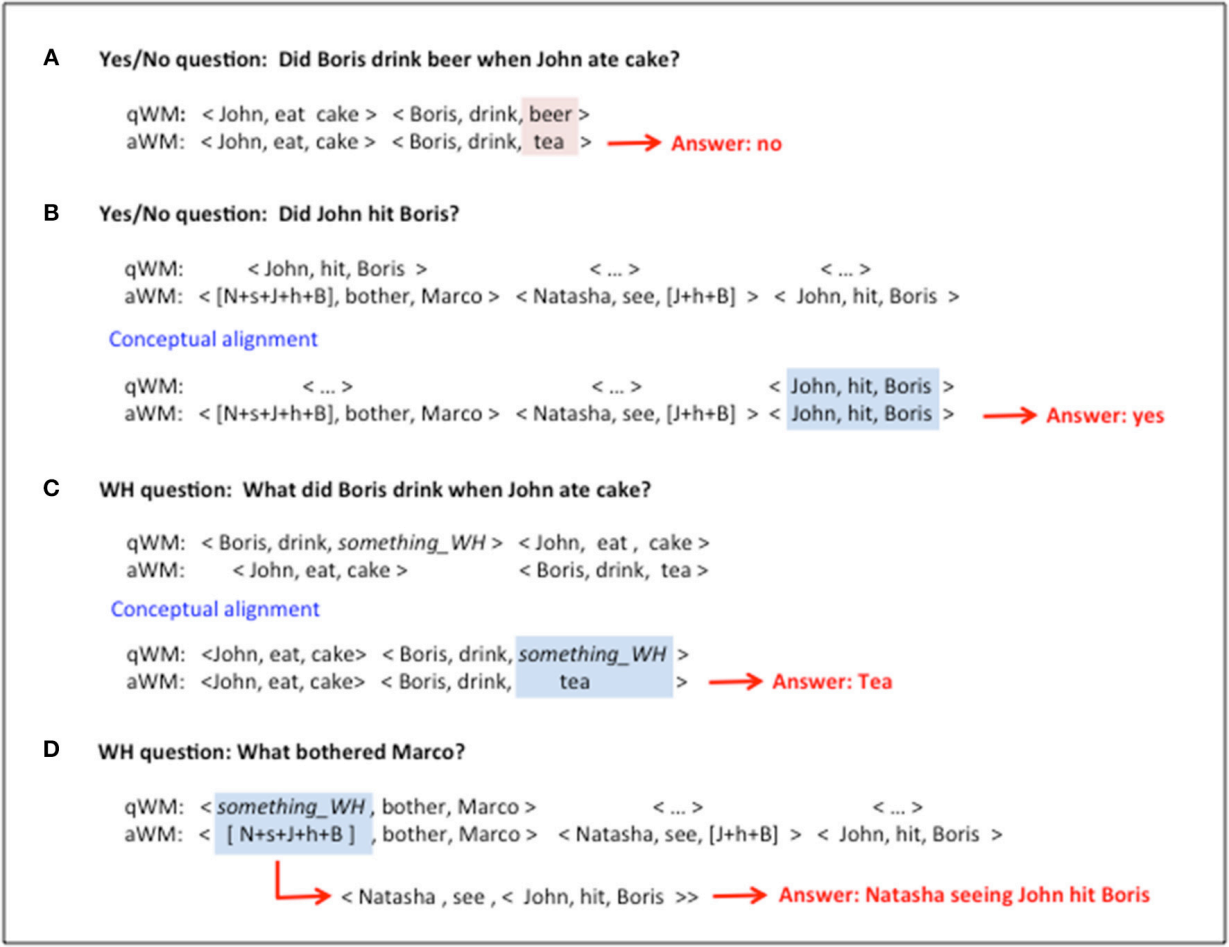
Question/Answers outcomes. **(A)** Simple Yes/No question with a negative answer due to a mismatch. **(B)** Yes/No question with pointers and alignment of information in the theta/gamma buffer (conceptual alignment). **(C)** WH question involving conceptual alignment. **(D)** WH question with answer containing pointers that need unpacking.

The next step is the second dechunking operation, which takes the spatially-coded triplets in the aWM buffer and produces a temporal sequence corresponding to the three elements of each triplet. This then allows an element-by-element interaction between elements of the question and elements of the stored memory, a process that can lead to the identification of the information necessary for matching the answer to the question. For example, for the question in Figure 3A, an element-wise comparison would reveal a mismatch between “beer” in qWM and “tea” that was retrieved in aWM, since the stored information is “John ate cake and Boris drank tea.” There are three possible outcomes for a yes/no question: (a) If the content in qWM matches the content of aWM, the answer is yes. (b) If at least one of the elements of the triplets in qWM and aWM are not similar, we consider there is no match and the answer is no, as in the example. (c) If there is a triplet in qSTM that does not synchronize with any triplet in aWM, there is no match and the answer is no. A more detailed description of the comparator is given in Mechanism 11.

After the comparator does its job, the answer is sent to the language generator to transform the triplet into a sentence in natural language, adding, for example, morphological inflection and adapting to the relevant register (Reiter and Dale, 1997). In the case of the question in Figure 3A this would result in a sentence like “No, *Boris drank tea*.” or just “*No.”*. In summary, the two dechunking operations allow a comparison of the question to the closest stored memory and the attainment of the desired information.

### Many Question Answering Situations Are More Complex

The example given above applies only to relatively simple sentences. In the following sections we discuss more complex cases.

#### Pointers

In the sentences discussed so far, there is no special relationship among triplets. They are just independent additive clauses. In other more complex sentences with subordinate clauses, triplets may be embedded inside other triplets, a relationship that may require a special mechanism for the relevant information to be stored. For instance, in the sentence “John saw Boris drinking tea” the main object, “Boris drinking tea,” is a complete proposition that requires its own triplet and cannot be represented as a cWORD. START addresses this complexity by establishing a formal dependence between triplets through the use of symbolic labels that link the main triplet to the embedded one. In START this is done by including the same digital address in two different elements (Katz, 1988). In the current example that would be “drink_2_” in the expression

**<John, see**_**1**_**, drink**_**2**_ **> < Boris, drink**_**2**_**, tea>**

with the meaning that John is seeing a drinking event that is the same event involving Boris and tea. Such symbolic labeling is essential to reconstructing the syntactic (and semantic) dependencies that characterize this sentence. However, in computer science the computational implementation of pointers using the same memory addresses often involves an arbitrary relation between positions (pointers) and their data contents, and assumes a hardwired ability to interpret a pointer, find the correct position in memory, and finally access the content. In the brain this is somewhat different since information is distributed and the associations between neurons are learned and carry content. For instance, for the Semantic Pointer hypothesis (Eliasmith, 2013) a pointer in the brain is a neural ensemble that carries a compressed (or reduced) representation of a much larger body of information. In this view, complex manipulation of concepts may be done at the level of semantic pointers and when more detailed information is needed it is retrieved by association based on content.

Here we explore this idea and the modularity of our network and propose a very simple alternative solution for syntactic structure storage. We consider that embedded triplets are represented using a compact representation that is stored in one of the elements of the main clause. This compact representation, that we refer to as a pointer, is synaptically linked to an embedded triplet stored in a different module and can be used operationally to find the embedded target. Conversely, because the compact representation does not resemble any known cWORD, this property can be used to recognize pointers in triplets. Pointers could then be implemented using operations like the superposition (sum) of the activation patterns of all the elements in the embedded triplet, or more complex operations (Stewart et al., 2014). In terms of these pointers the sentence “John saw Boris drinking tea” is represented by two triplets

**<John,see,[B+d+t]> <Boris,drink,tea>**

The pointer is the third element of the first triplet (denoted in the text by square brackets with the initials of the words of the embedded triplet and sum as an operator, [B+d+t]), while the embedded sentence is fully represented as the second triplet. It is the task of the parser to deduce the syntactic structure of the sentence and we do not describe here how this is done. We only assume that the parser consistently outputs the triplets in an order in which the content of a pointer appears always after (in a later gamma cycle) than the presentation of the pointer itself. It means that when we represent the sentence the pointer appears always to the left of the pointed triplet, as shown in the example above. The active neurons in the pointer become synaptically linked to the corresponding elements of the embedded triplet, a linkage that creates the necessary substrate for the retrieval of an answer to a question.

A similar and more generic proposal for binding representations of words with representation of syntactic roles is the Vector Symbolic Architecture (Gayler, 2003), a more abstract theory that proposes that brain manipulations of concepts is achieved by partially invertible algebraic operations (see different proposals in Smolensky, 1990; Plate, 2003; Stewart et al., 2011) on complex vector representation of words and roles. Although these operations allow embedded structures and could represent any complex sentence it is not completely clear what their precise neural correlates would be, which makes it difficult to test them in experimental setups (Eliasmith, 2013). Our proposal, on the other hand, results in clear predictions about spatial segregation of the triplet elements, compatible with (Frankland and Greene, 2015). Additionally, there will be regions that respond to multiple elements (i.e., the pointers), according to the structure of the target sentence (e.g., *John, hit* and *Boris* will all be able to activate the pointer that represents the event *John hit Boris* in the sentence *Marco saw John hit Boris*).

#### Word Order and Conceptual Alignment of qWM and aWM

The general mechanism involved in answering questions cannot rely on the assumption that the triplets in the question mirror precisely those in the episodic memories in terms of the number of triplets and their order. For instance, the question may refer to a specific subpart of a memory, resulting in a smaller sequence of triplets in qWM than in aWM. To deal with these cases, there needs to be a process that aligns the conceptually related triplets in the qWM and aWM buffers, a process that we refer to as conceptual alignment (see Mechanism 9). Imagine that the stored episodic memory is: “That Natasha saw John hit Boris bothered Marco,” and the question being asked is “Did John hit Boris?” Using the idea of pointers to represent embedded sentences, after successful retrieval of the memory the contents of the qWM and aWM buffers will be those in Figure 3B. Each triplet is held in a gamma slot in the aWM and/or qWM buffers. The theta-gamma buffers have ~7 available slots but we show only three of them in this example. Empty gamma slots are represented by <…>. Without alignment it will be impossible to detect that the first triplet in qWM corresponds to the last triplet in aWM since they are activated at different times. After conceptual alignment the buffers are as shown in the bottom half of Figure 3B. Now the matching contents are activated at same time and are synchronized (same gamma cycle in the qWM and aWM buffers). Therefore, the second dechunking can be applied and the resulting information sent to the comparator. For a plausible mechanism of conceptual alignment see Mechanism 9.

#### Answering WH Questions

WH questions, those which involve WH pronouns like *who, what, where*, and *when*, demand more than the simple comparison process that underlies YES/NO questions, by which successful matching between qWM and aWM elements results in *yes*, and *no* otherwise. For a WH question, a specific piece of information needs to be returned: the question buffer signals missing information and the answer buffer may contain the desired information. We consider that for a WH question the parser fills the element corresponding to the missing information with a special neural placeholder that indicates the category of the information being sought; this also is a marker that answering the question requires more than a simple matching operation. We consider that there are different kinds of neural placeholders, such as “*something_WH*” (for “what”), “*person_WH*” (for “who”), “*place_WH*” (for “where”), “*time_WH*” (for “when”), “*manner_WH*” (for “how”). Consider for instance the question “What did Boris drink when John ate cake?” in the context of “John ate cake and Boris drank tea.” After this question is parsed and stored into qWM as the triplets shown in the top half of Figure 3C, the result of retrieving the closest memory from eLTM and conceptually aligning the two buffers is shown in the bottom part of Figure 3C. At this point the contents of the buffers are sent, after the second dechunking operation, to the comparator. It evaluates if the found content (“tea”) is compatible with the class of possible answers indicated by the placeholder (“*something_WH*”). In the case of the example above the result is positive and the information is sent to the language generator that would produce a sentence like “*Boris drank tea*.” or just “*Tea.”*

But WH questions can involve more stages, especially in cases in which the memory has pointers. Consider for instance the question “What bothered Marco?” in the context of the sentence “That Natasha saw John hit Boris bothered Marco.” As before it is the task of the parser to recognize that the possible answers for this question encompass a broad category of events. Figure 3D shows the question encoded in the qMW and the retrieved memory in the aWM. Conceptual alignment is not necessary, and after resolving the pointer in the first triplet as the second triplet, and the pointer in the second as the third triplet, the answer is as shown in Figure 3D.

It is important to take into account the special nature of the representation of “*something_WH*.” In other words, the pointer “[N+s+J+h+B]” has to have some level of similarity with “*something_WH*” in a way that the brain knows that the question is answerable. Thus, if the question demanded a *where* type, we would not find a good overlap between “[N+s+J+h+B]” and “*where_WH*,” and so the answer is that the question is not answerable.

In sum the answer for the WH question can be achieved by the following recurrent steps:

1. After alignment, the element in aWM that corresponds to the placeholder is the answer for the WH question.
2. If the chosen element is not semantically meaningful (i.e., it is a pointer) there follows a search for an answer in the subsequent triplets in aWM, which corresponds to the triplet whose superposition of elements closely matches the pointer. We call this *unpacking* the pointer.
3. If the selected triplet itself contains a pointer, the process is iterated until all the pointers are unpacked.

The process of unpacking implies that there is a bidirectional interaction between the buffers and the comparator. In this process every time the comparator detects a pointer it allows new information relative to the pointer to be uploaded from the corresponding theta-gamma slot in aWM. Once all information is available, the language generator can produce the answer in natural language. This process is explained in Mechanism 11.

## Neural mechanisms

In this section we describe neurally realistic mechanisms that can implement some of the operations of the question answering system outlined above. Where possible, we use a mathematical description of the operations. In our model every word “w” is represented by a wordvector **V**(w) of N components, each component indicating the state of activation of a neuron in a network of size N. For the sake of simplicity, we consider that the basic units, the subregions that represent each element of triplets, are all size N networks in both WM and eLTM. Another simplifying assumption is that the wordvector that represents a given word, for instance “dog,” is rigorously the same (has the same firing pattern) in all networks involved in the processing or storage of the concept dog. We also assume that the representation is sparse in the sense that only a small fraction of the N neurons available in the network is used to represent a given word.

### Mechanism 1: Compositional Coding

Compositional coding is important in our scheme because it reduces the number of triplets needed to encode a sentence. Elements can encode not only nouns and verbs, but also modifiers (adjectives/adverbs) along with other predicates and arguments. Whereas, in the simplest case, a word might be represented by binary firing rate patterns in the N cells, using a compositional code, additional information can be stored by making rates continuous. Such a coding scheme has been observed in the hippocampus and called rate-remapping (Leutgeb et al., 2005) and has been modeled in (Rennó-Costa et al., 2010). Thus, cells that are active (at any level) define linguistic entities such as nouns and verbs, whereas their rates encode modifiers like adjectives and adverbs. We assume that these entities can be represented by wordvectors (Baroni and Lenci, 2010; Mikolov, 2014) and their modifiers can be implemented by a matrix operation (Baroni and Zamparelli, 2010) that changes the relative values of the wordvector elements

V(noun+adj)= A(adj)·V(noun)

where the matrix **A**(*adj*) implements the adjective, “*adj*.”

### Mechanism 2: Working Memory Buffers

We assume that WM is a network of size 3N and that it can actively hold any sequence of 7 triplets in a theta-gamma code. Details of how this coding scheme can be neurally implemented can be found in (Lisman and Idiart, 1995; Lundqvist et al., 2011) or (Koene and Hasselmo, 2007).

### Mechanism 3: Long Term Memory

Each module in eLTM is a standard attractor network that stores a number of memories in terms of modifiable synaptic recurrent connections. Each attractor defines a given triplet. A stable pattern is given by the generic expression

$$\begin{array}{l} V^{triplet}=\;RNN\left(\;I^{triplet},\;I^{other\;triplets},I^{qWM}\right) \end{array}$$

where RNN(I) is a function that implements a recurrent neural network, *I*^*a*^ = *W*^*ab*^·*V*^*b*^ are the inputs arriving in network “a” due to the activity in the network “b,” and *W*^*ab*^ is the *N*×*N* synaptic connection matrix from network “b” to “a.” Details on how attractors can be neurally implemented can be found in (de Almeida et al., 2007; Lundqvist et al., 2011; Rennó-Costa et al., 2014).

### Mechanism 4: Pointers

**Figure 4:**
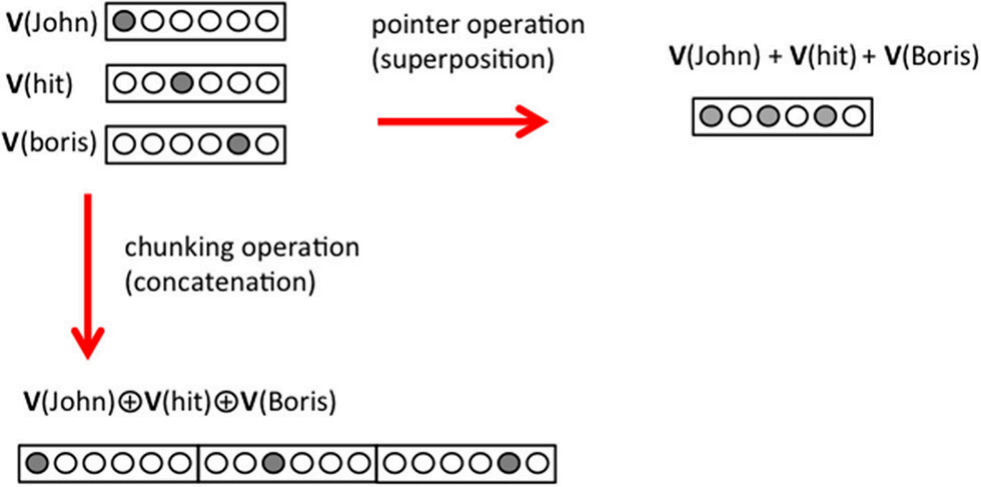
Pointer operation and chunking operation. In the framework of this paper, a pointer is built from the superposition of the neural representation of the elements of a triplet (top right corner). In the chunking operation on the other hand the neural representations of the elements are concatenated in a larger representation (bottom left corner). For simplicity, we represent a concept with a single active neuron (gray circle) but we consider that many neurons are involved in the representation of a concept.

We propose that a pointer is built by the superposition of the wordvectors of the triplet it points to. For instance, in the case of the sentence “Natasha saw John hit Boris,” the element in the object position, “John hit Boris,” cannot be written as a cWORD using compositional code. Therefore, a pointer must be used and in term of triplets the sentence is shown in Figure 4. < Natasha, see, [J+h+B] > <John, hit, Boris >. We consider that, in vector form, the pointer [J+h+B] is defined as

V([J+h+B])= V(John)+V(hit)+V(Boris)

where the operation “+” adds the pattern of activity of the different concepts. It means that the neurons that are active in the representations of “John,” “hit,” and “Boris,” will be all active at same time in the representation of [J+h+B] (Figure 4). An expected property of the pointer is that it does not have a representation in semantic memory and therefore cannot be interpreted by the language generator. Later on we use this property when processing the answers of a question (see Mechanism 11); if an element is not interpretable, it is a pointer and the triplet it points to has to be retrieved and the information unpacked.

Both the operations of code composition and of pointer creation occur within the parser. Therefore, we do not describe the underlying neural mechanism.

### Mechanism 5: First Chunking

Chunking is a process of concatenation of wordvectors in a larger spatial representation. The words “subj,” “verb,” and “obj” that were represented by wordvectors **V**(subj), **V**(verb), and **V**(obj) activated at different times in a network of size N, are represented in specific subregions of a size 3N network after chunking

$$\begin{array}{l} \text{T}\left(<subj,verb,obj>\right)=\;\text{V}\left(subj\right)⊕\text{V}\left(verb\right)⊕\text{V}\left(obj\right) \end{array}$$

where now **T** is a wordvector of a triplet (see Figure 4). This process corresponds to the first chunking stage. We assume that the first chunking operation happens within the parser and we do not model it explicitly. At this point, the parser might need to route the different elements of the triple to different brain subregions (Vigliocco et al., 2011; Murphy et al., 2012), and this can be achieve by different cortical circuits (Akam and Kullmann, 2010; Palmigiano et al., 2017).

### Mechanism 6: Second Chunking

The second chunking occurs when the triplets of an episodic memory (or sentence) are concatenated into a completely spatial structure of size 7^*^3N neurons

$$\begin{array}{l} E\left(triplet_{1},\cdots ,\;triplet_{7}\;\right)\\ \;=\;T(triplet_{1})⊕\;T(triplet_{2})⊕\;\cdots ⊕T(triplet_{7}) \end{array}$$

This process happens between the WM buffers and the eLTM network. A sentence held in WM, composed of up to 7 triplets kept in different gamma slots, is laid out in order in different spatial subregions in eLTM. A possible network mechanism for such a process has been proposed in (Sanders et al., 2014). The idea is that the different subregions have bistable neurons with slightly different properties, such that when information arrives only one subregion will be available to store it, while the others are locked. Once a pattern is established in a given subregion, this subregion becomes unavailable to the new incoming information (by inhibitory feedback), while at same time excitatory connections enable the next subregion to store the new input.

Once the triples are loaded into the eLTM, the patterns should become persistent by some mechanisms of memory consolidation. The organization of patterns in gamma frequency cycles provide ideal timing for triggering long term plasticity (Jensen and Lisman, 2005).

### Mechanism 7: Retrieval From eLTM

Triplets are stored in different gamma slots in qWM buffer and are used individually to stimulate all the modules of eLTM. For simplicity we consider that each neuron in qWM is connected with a single neuron in each module of eLTM, and that conversely each neuron in eLTM receives a single input from qWM. Therefore, every time a triplet is active in qWM, neurons correspondent to this triplet in all eLTM modules receive inputs through synaptic connections. We call this information broadcast.

A module in eLTM works as an associative memory that stores memories of triplets as dynamic attractors. If a broadcasted triplet is close to a particular memory in a given module, this memory is activated in this module (as an “attractor”). But if the module already has an active memory, the memory evoked by the new triplet has to compete with the pre-existing attractor. A new memory can only extinguish a pre-existing attractor if the new memory has an advantage, for instance, if it has been primed (see below) by a previously broadcasted triplet; otherwise the pre-existing attractor wins the competition. Competition can be mediated by feedback inhibitory circuits that drive the gamma frequency oscillation (de Almeida et al., 2009).

The priming process is a novel proposal about how associative networks might function. In our model, priming occurs via learned connections between modules. To see the importance of priming, consider what happens after the first query activates all the modules that contain a triplet. If associative interactions across the modules could excite other elements/triplets, all associated sequence memories (e.g., sentences) would be activated. But these would also have associates, leading eventually to activation of a large fraction of cells. The priming operation solves this problem by not yielding direct excitation of associates, but preparing them for possible future activation. As a consequence, the influence of priming is felt because if a primed cell is activated during the query of a second (or later) triplet, it becomes active and can outcompete other non-primed triplets. At the end of the query process in which all triplets of the qWM have already been processed, activated cells in the eLTM network will represent the maximal *overlap* with the qWM. This is important because the maximum overlap condition defines the memory that best matches the question posed.

We found that while priming worked excellently to find the best overlap, it doesn't produce activation of the parts of a memory that were not queried (recall that the question may be shorter than the stored memory). However, activating the whole memory is useful. We therefore posit that at the end of the query process, the synaptic function is changed from a mode in which synapses can only prime targets to one in which they can excite targets. Such a change might be implemented physiologically by making the priming process use only NMDA receptors at associative synapses; such receptors don't activate postsynaptic cells, but can make them more activatable by other inputs (Vargas-Caballero and Robinson, 2004; Iacobucci and Popescu, 2017). A neuromodulatory process at the end of querying might then allow AMPA receptors to also function at these synapses. When AMPA receptors are functional, presynaptic activity leads to activation of target cells, thereby allowing the full memory to be activated by the parts already activated during the query process (Sasaki et al., 2011).

### Mechanism 8: First Dechunking

Once a stable pattern is obtained in eLTM, the inputs coming from different modules of eLTM are routed to the aWM one at the time, following the order in which they are spatially stored. The aWM buffer absorbs this information using the theta-gamma code. This sequential routing might be accomplished by a traveling wave in the eLTM network. More work will be necessary to determine how this might work. The ability of a buffer to absorb sequentially input memories and make them active in sequential gamma cycles is demonstrated in (Lisman and Idiart, 1995).

### Mechanism 9: Conceptual Alignment

**Figure 5:**
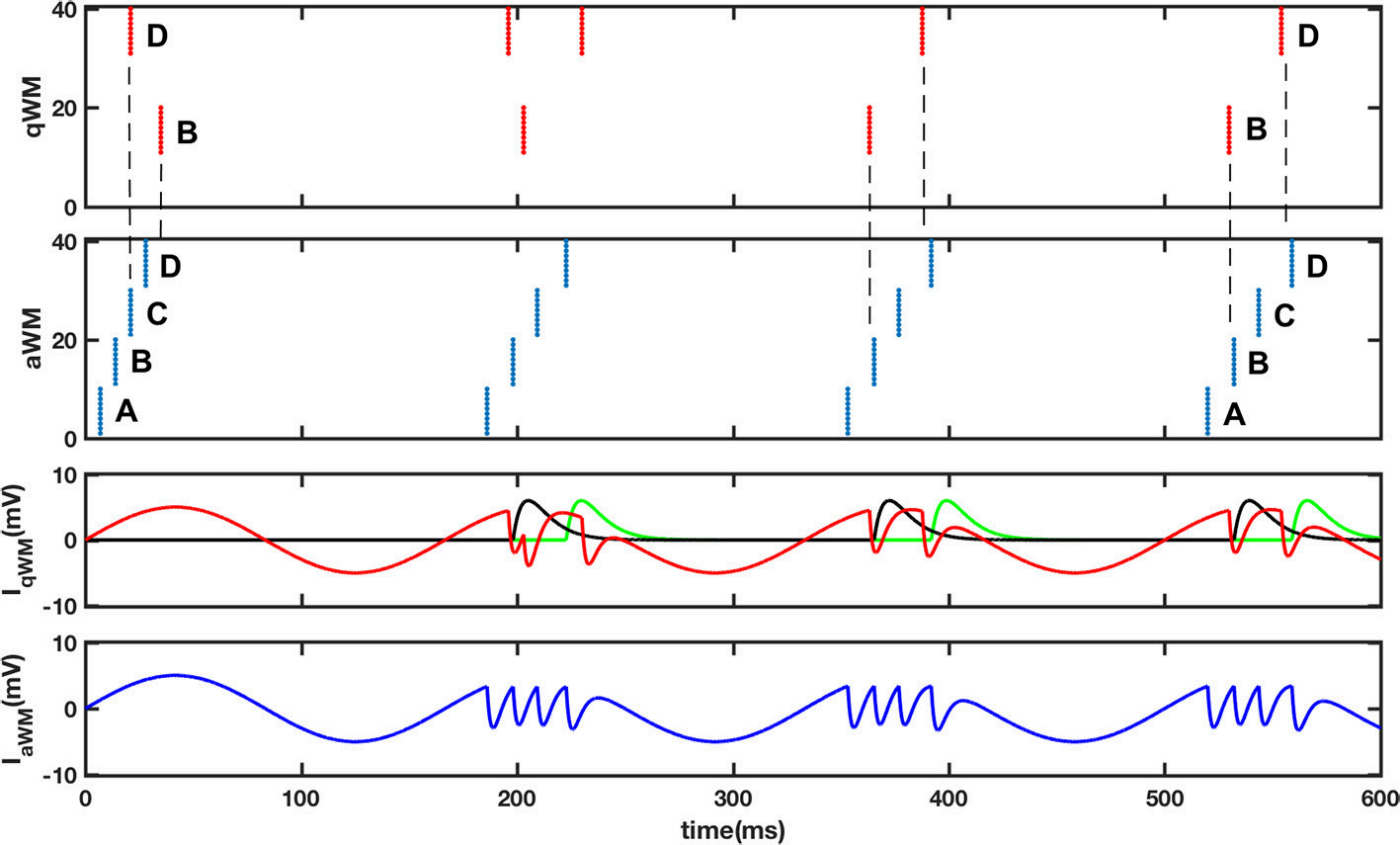
Synchronization of the question buffer (qWM) and the answer buffer (aWM). The top 2 panels display raster plots of 40 neurons in qWM (red) and aWM (blue), representing four different patterns. Initially patterns B and D in qWM and aWM are in different gamma slots (see dashed lines). Due to the inputs coming from the aWM network (neuron “*i*” in aWM excites neuron “*i*” in qWM, and all other neurons in aWM make inhibitory connections to neurons in qWM) the neurons in qWM synchronize with the corresponding neurons in aWM. Note that the alignment can work even when there are empty slots (in the end B and D are 2 gamma cycles apart). The bottom 2 panels indicate the inputs each neuron in the buffers receives. In the inputs to qWM neurons the red line represents the theta input plus the feedback inhibition, while the black and green lines represent inputs coming from the aWM buffer. In the last panel the blue line represents the inputs to aWM neurons where it is possible to see the gamma slots corresponding to four active memories. Two patterns D and B are active in qWM while in aWM four patterns are active A,B,C, and D. The buffers are as described in Lisman and Idiart (1995) with the following difference A_ADP_ = 6, 7 mV, *t*_ihn_ = 3 ms and with excitatory connections from aWM to qWM with A_exc_ = 6 mV and *t*_exc_ = 7 ms, and inhibitory connections from aWM to qWM with A_inh_ = −2 mV and *t*_inh_ = 3 ms.

An inevitable requirement of the dual buffer system for question and answer is the need for synchronization. Our proposal is that the aWM buffer uses excitation to synchronize the qWM buffer in a way that the brings the conceptually most similar triplets to the same theta phase, i.e., the same gamma slot. We assume that the excitatory connections between aWM and qWM are learned during encoding of a memory in eLTM. Figure 5 illustrates a possible implementation of the synchronization process using the original theta-gamma model described in (Lisman and Idiart, 1995). The details of the model and the parameter values are described in the figure caption. In the simulation both qWM and aWM buffers are identical networks of N neurons, whose intrinsic after-depolarization mechanisms associated with an external theta input allow working memory maintenance by the continued activation of previously tagged neurons in subsequent theta cycles. Feedback inhibition between different ensembles provides memory segregation in different gamma slots. We adopt the simplifying assumption that there is a correspondence among neurons such that the first neuron in one buffer corresponds to the first neuron in the other buffer, and so on. Neurons in qWM receive excitatory connections from corresponding neurons in aWM, and inhibitory connections from all other neurons in aWM. In the two top panels of Figure 5 the process of alignment is displayed; in the first theta phase (used as initialization of the network), there are 2 triplets in the qWM buffer, each represented by a set of 10 neurons firing synchronously (red dots) while there are 4 triplets in the aWM (blue dots). The neural patterns are indicated by letters. The problem that needs correction is that patterns B and D are firing in different slots in the two buffers. The figure shows what happens during 4 theta cycles. The bottom two panels show the input current that typical active neurons in qWM (red line) and in aWM (blue line) receive. The black and green lines are the excitation that neurons in patterns B and D in qWM receive from neurons in patterns B and D in aWM. Network dynamics automatically rearranges the corresponding neurons firing in the qWM so that the gamma slots of corresponding items in the qWM buffer become aligned with those in the aWM. Although Figure 5 illustrates the process of alignment using a very simple model, a more comprehensible and physiologically testable model could be accomplished following the approach in (Lundqvist et al., 2010).

### Mechanism 10: The Second Dechunking

Second dechunking is a key issue that needs to be described. To examine the content of elements and their semantic associates, it is critical that only one element be active at time; only in this way can semantic information be unequivocally linked to specific element in triplets. There have been no prior models of how a spatial pattern can be converted into a temporal pattern, but the following general outline seems feasible. When a given triplet is active in a low frequency gamma cycle (e.g., 30 Hz) information from the aWM buffer is sent to a buffer that operates at 3 times the frequency, i.e., 90 Hz. During the first high frequency cycle, a router takes the first element of the triplet and puts the information in the first slot of the high frequency buffer. Then during the second high frequency cycle, the information from the second element is routed and stored in the second high frequency slot. The general implication is that the brain may contain WM buffers in which gamma frequencies differ by integral multiples.

### Mechanism 11: The Comparator

The comparator receives simultaneous elements from qWM and aWM. It works in two modes. The **Y/N** mode is necessary to solve yes/no questions. The operation performed by the comparator is a subtraction that is akin to a subsumption relation (Carpenter, 1992). It detects similarity between the elements coming from the two buffers by using inhibitory inputs from aWM to cancel inputs from qWM. The subtraction only applies for gamma slots that are filled both in qWM and aWM. No comparison is made when empty gamma slots are present. If this condition is satisfied and the inputs do not cancel out, a residual activity is detected in the comparator. This residual is sent to a network downstream, indicating that the answer is “no,” otherwise, if the difference results in no residual activation, then the answer is “yes.”

The **WH** mode is necessary to answer WH questions. The same process of subtraction is performed but now the residual activity carries the information necessary to answer the WH question. In particular, it is the goal of that evaluation to access if the answer in aWM fulfills the category of the placeholder in qWM. If the result is positive that information is passed on to the language generator otherwise the answer is “I do not know.”

One complication is that the selected element may not correspond to any concept in semantic memory and instead be a pointer. If so, the triplet to which the pointer points must be identified, **unpacked** and examined in order to answer the question. Consider the sentence “*That Natasha saw John hit Boris bothered Marco*,” and the question “What bothered Marco?”. The answer obtained after alignment and comparison indicates the element: “[N+s+J+h+B],” Figure 4D. Since it is a pointer it must be unpacked by finding and processing the triplet to which the pointer points. The triplet < Natasha, see, [J+h+B] > is the result as it is the one whose superposition is the closest match to the pointer. So now instead of sending [N+s+J+h+B] to be lexically realized, we now send three elements: “Natasha,” “see,” and “[J+h+B]” one at a time. But again we face a problem with “[J+h+B],” so it is necessary to go back to the triplets in the aWM and retrieve the closest match to “[J+h+B]”. This time the triplet with “John,” “hit,” and “Boris” is selected and sent. Now all elements are recognized as words and the answer is the syntactic combination (corrected for tense and number, etc…) and lexical realization of the words “Natasha,” “see,” “John,” “hit,” and “Boris” (Reiter and Dale, 1997).

The comparator operates in semantic memory to obtain all the relevant information needed for the comparisons.

### Mechanism 12: Semantic Memory

The implementation of semantic memory (SM) will be important for making a robust question-answering system. We point here to some of the functionalities required. The parser will have to determine whether two words are similar enough to be both represented by a cWORD. Some sentences may also involve descriptions, paraphrases, synonyms or properties of the words in a stored memory that cannot be dealt with by cWORDs. For instance, we may refer to *John* as the *Brandeis professor*, or as *Natasha's husband*, or using any of the properties that are applicable to him by virtue of being a man, a person, or a living being. Knowledge about meaningful semantic elements is stored in a semantic memory (Jones et al., 2015). SM is a large network that contains information about concepts, their taxonomic connections and semantic relations. We assume that concepts are represented as sets of abstract feature values that describe their relevant properties (like height, shape, age and speed). Elements may be organized taxonomically and be semantically related to one another by various relations, such as by hypernymy or superordination relation (man *is a* person), synonymy (child and kid) and meronymy or part-to-whole relation (door *is part of* house) (Fellbaum, 1998). In SM, the similarity of two concepts can be measured by the overlap between their neural assemblies. Concepts that are very close share many neurons, as they have many feature values in common (Andrews et al., 2008; Vigliocco et al., 2011; Fyshe et al., 2014). Relatedness on the other hand does not imply similar neural assemblies but neural assemblies that are strongly connected. An example of similarity would be “chimpanzee” and “primate” and an example of relatedness would be “chimpanzee” and “tree” (Lin, 1998; Herdagdelen et al., 2009).

## Conclusions

In this work we presented a list of core concepts that could underlie the neural realization of an intelligent system capable of storing sentences and answering questions about the content of these sentences. The general ideas about the functionalities and operations needed for answering a question are based on the workings of START an AI that is completely non-neural in implementation. Having now shown how core concepts in START could be neurally implemented, it is useful to review the types of neural building blocks that we posit are needed for this implementation.

1. Working memory buffers or theta-gamma buffers that hold input/output information.
2. Attractor neural networks that store complete sentence information as spatial patterns.
3. The idea that networks can have spatial subregions dedicated to the storage of particular types of information.
4. Neural codes capable of very rich information storage such that nouns/verbs are represented by the particular cells that are active whereas the related adjective/adverb is coded by modulation of the firing rate of these cells. This coding scheme is related to the observed process of rate-remapping in the hippocampus. In the hippocampus, however, an ensemble of equivalent cells represents a place and are then modulated by rate to encode sensory features. A still richer coding scheme would make the ensemble encoded by non-equivalent cells that represent different features capable of representing a family of semantically related concepts.
5. Chunking converts temporal patterns into spatial patterns.
6. Dechunking coverts spatial patterns into temporal patterns. Cortical waves may be important for this process.
7. The above two processes require temporally controllable routers.
8. We suggest that the second dechunking could be implemented by coupling an oscillator at 3x frequency to one at 1x frequency.
9. Priming facilitates the search process by which the stored memory most closely related to the query is found. We suggest that priming is due to synapses that contain the NMDA type of glutamate receptor, but not the AMPA type.
10. To achieve the final winner-take-all process that selects the stored memory closest to the query, AMPA channels become functional at synapses that were previously NMDA only.
11. In order to find the correct elements in the retrieved memory the qWM and aWM buffers have to undergo conceptual alignment, where similar triplets in both buffers synchronize in the same gamma slot. The alignment in implemented as excitatory and inhibitory connections between the two buffers.
12. The pointer mechanism for representing the complex hierarchical phrasal structures characteristic of language in the simpler modular structure proposed for WM and eLTM.

These 12 properties have varying degrees of experimental support but all seem within the realm of what the brain can do. Some of these processes have been directly analyzed by neural simulation that confirms their functionality. Others have not been analyzed by simulation and should be the subject of further research. At the start of our effort it was unclear what neural computations were required and whether these could be neurally implemented. Having studied how to implement question answering, our general conclusion is that known or plausible neural mechanisms are sufficient to perform this complex cognitive function. Although individual components of the architecture have already been computationally implemented (Lisman and Idiart, 1995; Jensen and Lisman, 2005; de Almeida et al., 2007; Sanders et al., 2014) the development of a complete functional architecture is left for future work. Additionally, further reinterpreting the model proposed in this work in terms of potential interactions with other systems such as the visual and auditory systems would provide additional sources of information for disambiguating memories during storage and retrieval (Howard et al., 2005). Moreover, by considering the viewpoint of embodied cognition we would obtain other neural mechanisms to support language processing combining information from multiple sources such as vision and hearing (Zwaan, 2008).

## Author Contributions

JL and MI conceived of the presented idea, and proposed the neurobiological models. BK proposed the original AI model. AV and BK contributed with the linguistic background. JL, MI, AV, BK, and CR-C discussed the results and contributed to the final manuscript.

### Conflict of Interest Statement

The authors declare that the research was conducted in the absence of any commercial or financial relationships that could be construed as a potential conflict of interest.
